# *In silico* characterization of cysteine-stabilized αβ defensins from neglected unicellular microeukaryotes

**DOI:** 10.1186/s12866-023-02817-w

**Published:** 2023-03-25

**Authors:** Marcus Vinicius Xavier Senra

**Affiliations:** Centro de Ciências Naturais e Humanas, Universidade Federal do ABC, Santo André, São Paulo, 09210-580 Brazil

**Keywords:** Ciliophora, Genome mining, Antimicrobial peptide, Bioprospection

## Abstract

**Background:**

The emergence of multi-resistant pathogens have increased dramatically in recent years, becoming a major public-health concern. Among other promising antimicrobial molecules with potential to assist in this worldwide struggle, cysteine-stabilized αβ (CS-αβ) defensins are attracting attention due their efficacy, stability, and broad spectrum against viruses, bacteria, fungi, and protists, including many known human pathogens.

**Results:**

Here, 23 genomes of ciliated protists were screened and two CS-αβ defensins with a likely antifungal activity were identified and characterized, using bioinformatics, from a culturable freshwater species, *Laurentiella* sp. (LsAMP-1 and LsAMP-2). Although any potential cellular ligand could be predicted for LsAMP-2; evidences from structural, molecular dynamics, and docking analyses suggest that LsAMP-1 may form stably associations with phosphatidylinositol 4,5-bisphosphates (PIP2), a phospholipid found on many eukaryotic cells, which could, in turn, represent an anchorage mechanism within plasma membrane of targeted cells.

**Conclusion:**

These data stress that more biotechnology-oriented studies should be conducted on neglected protists, such ciliates, which could become valuable sources of novel bioactive molecules for therapeutic uses.

**Supplementary Information:**

The online version contains supplementary material available at 10.1186/s12866-023-02817-w.

## Background

The discovery and therapeutic use of antimicrobial agents represent one of the greatest milestones in medical science, contributing to saving countless lives every year [1]. Unfortunately, only 7 years were required, since penicillin introduction [2], for the emergence of an antibiotic-induced resistant strain (*Staphylococcus aureus*), which were isolated from hospitalized patients in London in 1948 [3]. Afterwards, countless preoccupying cases of resistant and multidrug-resistant microorganisms, such as *Mycobacterium tuberculosis* [4], *Streptococcus pyogenes* [5], *Staphylococcus aureus* [6], *Enterobacter cloacae* [7], *Pseudomonas aeruginosa* [8], *Candida albicans*, and *Aspergillus fumigatus* [9] started to be reported, resulting from natural selection processes, but exacerbated from the misuse and overuse of antibiotics [2]. Therefore, the development of new antimicrobials is utmost necessary to combat this major concern in public health.

One promising class of molecules is the cysteine-stabilized α-helical β-sheet (CS-αβ) defensins. These antimicrobial peptides (AMPs) are typically small (34–54 amino acid residues), amphipathic (defined hydrophobic and hydrophilic regions within the same molecule), mostly cationic, and cysteine-rich defensins that adopt a canonical CS-αβ scaffold, consisting in an α-helix and two β-strand antiparallel β-sheet stabilized by two disulfide bridges linking the α-helix to the C-terminal β-strand, and a third bridge connecting the N-terminus to the first β-strand [10].

Critical players of humoral defense systems of fungi [11], plants [12] and some invertebrates [13, 14], these AMPs may exert their functions through a diversity of mechanisms of actions, including membrane disruption and pore formation [15], ion channels blockage [16, 17], and interference to different intracellular pathways [18–20]; and are highly effective against a broad spectrum of pathogens [21]. While plant CS-αβ defensins are predominantly antifungal [21], with a few insecticidal [18] and bactericidal [21]; fungal and animal CS-αβ defensins are mostly antibacterial [21], with some reports of antiprotozoals [22, 23] and fungi [13, 24, 25].

Here, 23 publicly available genomes of ciliated protists, an ubiquitous and highly diverse group of unicellular microeukaryotes [26], were screened for CS-αβ defensin homologs and possible cellular ligands are presented.

## Results

Screenings for CS-αβ defensin homologs within the predicted proteomes of 23 ciliate species were performed using a combined approach, which consisted in pairwise sequence alignments against SwissProt database and profile search, using a previously described CS-αβ defensins cysteine distribution pattern that is conserved across CS-αβ defensins [27]. After a series of filtering steps (see methods for details), two unannotated CS-αβ defensins were identified from the genome of the freshwater hypotrichous ciliate, *Laurentiella* sp. (LsAMP−1 and LsAMP−2) and data are summarized in Table 1.

**Table 1:** Basic sequence information and antimicrobial activity prediction of ciliate defensins

					Antimicrobial activity pred.		
Defensin	Host (accession number)	Scaffold [position]	Sequence (aa)†	Swiss-Prot Best hit (%ID)	ADAM	CAMP‡	iAMP-2 L
LsAMP-1	*Laurentiella sp. (GCA_001272975.2)*	LASS02013305.1 [977 - 771]	MVFSKSFLLTLYFFIATILLNLVQA**DVDVGSCFVFLPDYRSDCNGYCQQRGYKGGHCGSIFNVKCWCET**	Heliomicin (64%)	1.48	0.867	Antifungal
LsAMP-2	*Laurentiella sp. (GCA_001272975.2)*	LASS02014383.1 [1468 - 1070]	MSINSQIRVLVLFVFLVLTYINQVSA**DVLIGSCVWGAVDYKSDCSGYCESRGYSGGHCGSFGNVDCWCNVDE**	Heliomicin (76%)	1.93	0.975	Antifungal

† Sequence identity (ID) and ‡ coverage (cov) ‡ between query/template

**Fig. 1:**
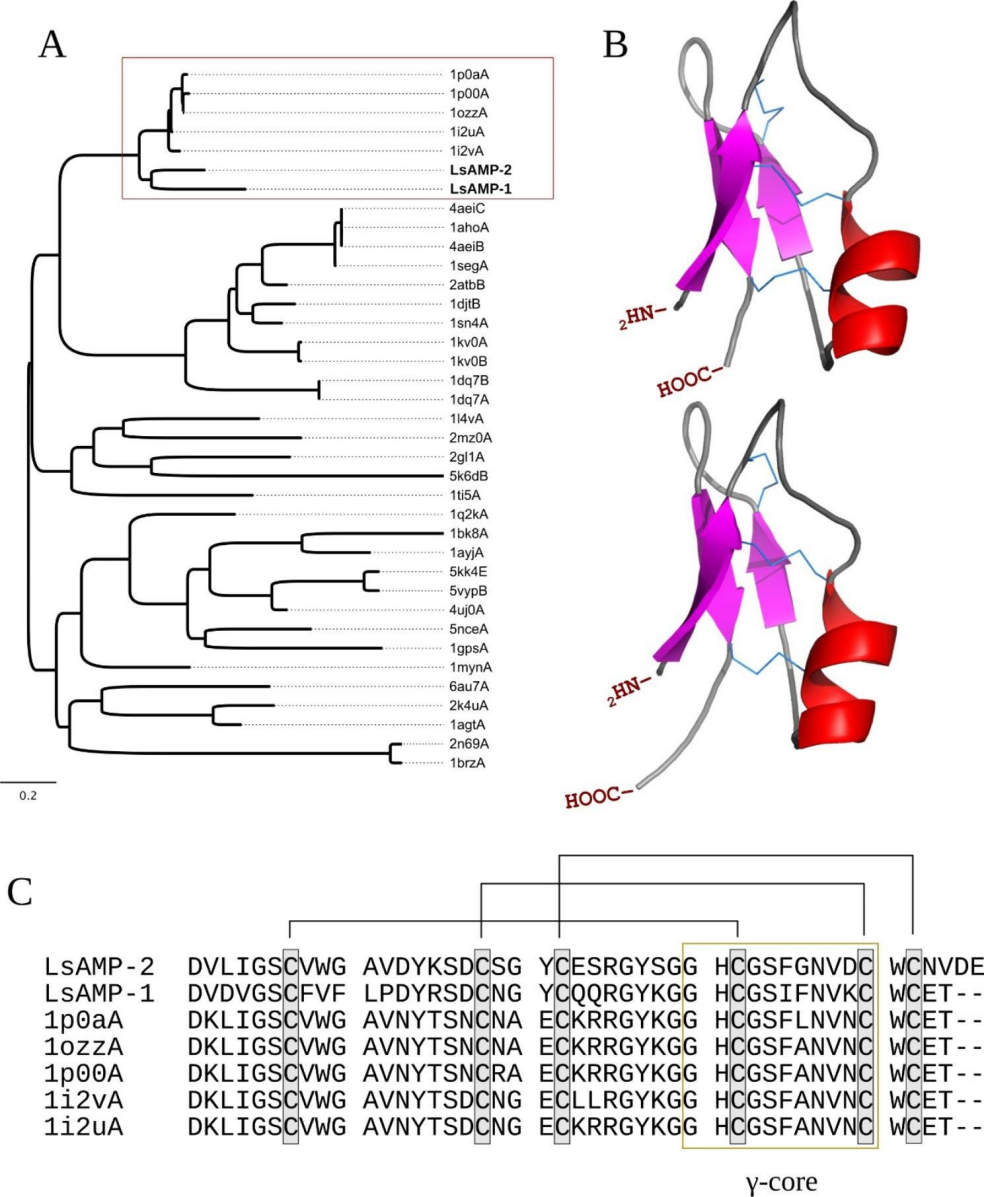
(**A**) Neighbor-joining phylogenetic tree inferred from structural alignment of proteins available from PDB. LsAMP-1 and LsAMP-2 are in bold. The red square highlights the monophyletic cluster including LsAMP-1, LsAMP-2, heliomicin (1i2vA and 1i2uA) and ARD1 (1p0aA and 1p0oA). (**B**) Theoretical three-dimensional models of LsAMP-1 (top) and LsAMP-2 (botton). Disulfide bridges are connected with sticks. And (**C**) alignment of defensin mature sequences highlighted in (**A**). Lines in black represent the cysteine connections and the γ-core of these proteins are within the yellow square

LsAMP−1 and LsAMP−2 are both anionic (net charge of−1 and−5 at pH 7,0) with N-terminal signal peptides spanning through the first 20 amino acid (aa) residues and mature sequences of 44 and 46 aa residues, in which contains a conserved invertebrate defensin domain (IPR001542) (Table 1). These defensins share a 61% of sequence identity and are highly similar to previously characterized lepidopterans antifungal CS-αβ defensins, heliomicin (PDB:1i2u/1i2v), isolated from *Heliothis virescens* (Noctuidae) [24] (64% and 76% of identity); and ARD1 (PDB:1p0a/1p0o), isolated from *Archaeoprepona demophon* (Nymphalidae) [28] (59% and 71% identity) (Table 1; Fig. 1).

LsAMP−1 and LsAMP−2 theoretical structures were predicted through comparative modeling, using heliomicin and ARD1 as templates, respectively (Fig. 1b; Table 2), both consisting of an N-terminal β-strand followed by an α-helix and two antiparallel β-strands (βαββ) scaffold, stabilized by three disulfide bridges (Cys^1^-Cys^4^, Cys^2^-Cys^5^, and Cys^3^-Cys^6^) (Fig. 1b-c). At their C-terminus there is a canonical signature (Gly-X-Cys-X_3-9_-Cys, where X represents any residue) recognized as the γ-core, which plays a role in the antimicrobial activity of other CS-αβ defensins [29, 30] (Fig. 1c).

**Table 2:** Template information and quality check of ciliate defensin 3D models

					Ramachandran plot analysis			
Defensin	Template	Template classification	ID†	Cov‡	Residues in favored regions (%)	Residues in allowed regions (%)	Outliers	ProSA (Z-Score)
LsAMP-1	1i2uA	Antifungal	0.64	1	92.9	100	-	-5.43
LsAMP-2	1p0aA	Antifungal	0.71	0.97	90.9	100	-	-5.74

† Sequence identity (ID) and ‡ coverage (cov) ‡ between query/template

**Fig. 2:**
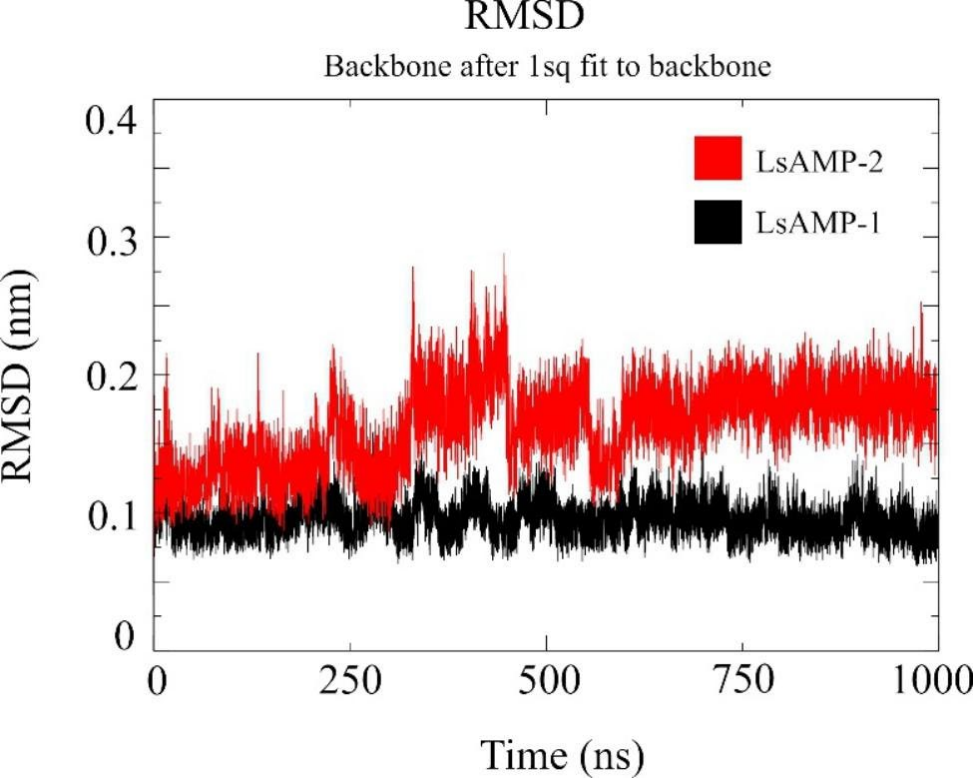
Molecular dynamic evaluation of LsAMP-1 and LsAMP-2 theoretical structures. (**a**) A root mean square deviation (RMSD) plot, showing the mean backbone variation over the 1µs of this simulation

All amino acid residues from these models emerged within favorable and acceptable regions of Ramachandran plots (Table 2). Analyzes of root mean square deviations (RMSD), root mean square fluctuations (RMSF), and dictionary of protein secondary structure (DSSP) indicate that movement of amino acid residues are within acceptable ranges, most of them occurring at the carboxy terminal (Fig. 2) and secondary structures remained almost unchanged along the entire MD simulation (Additional file 1), evidencing the quality and stability of these models.

**Fig. 3:**
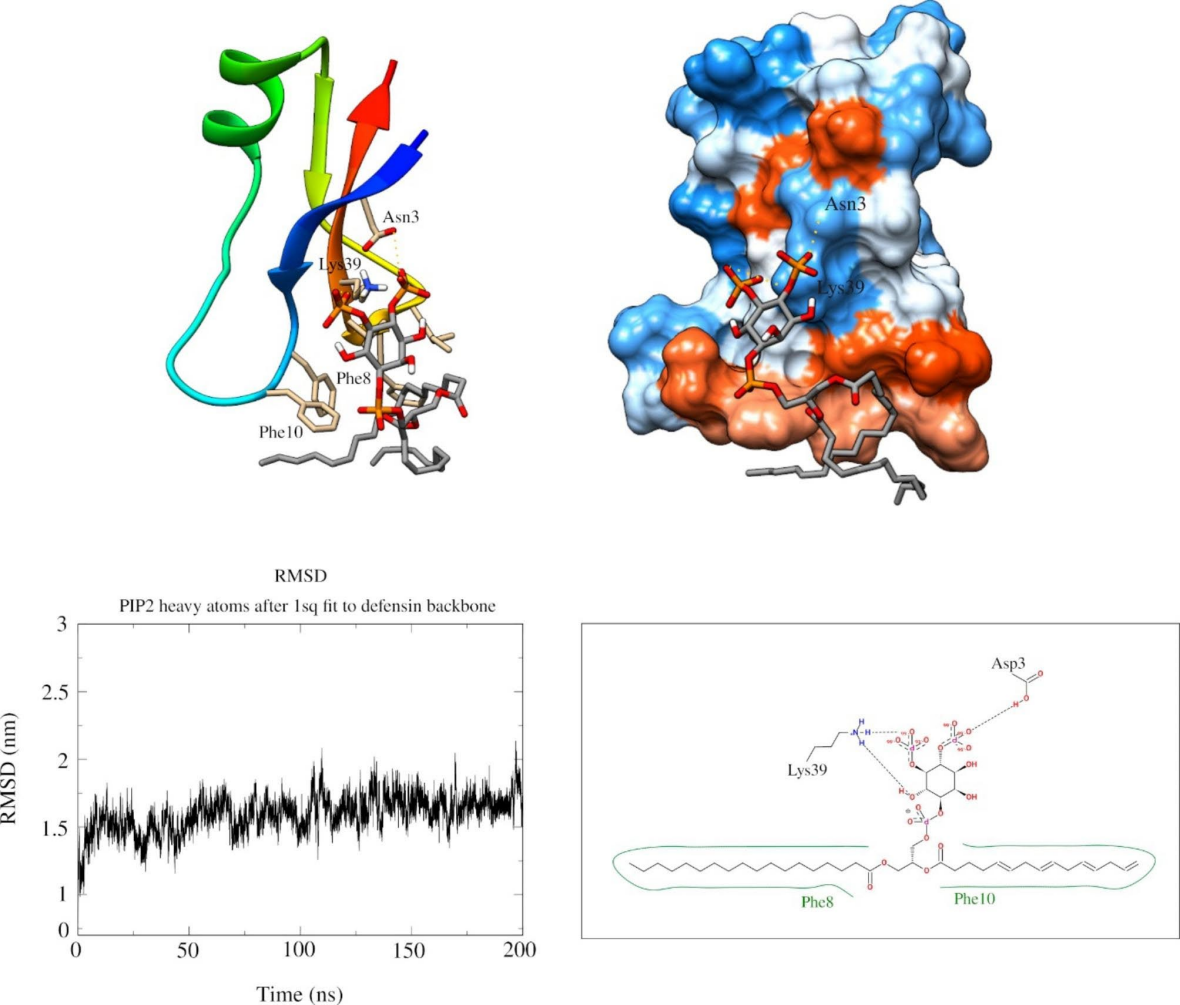
LsAMP-1 may stably interact with PIP2. The top-most part of this figure shows 3D representations of LsAMP-1-PIP2 binding pose at the last frame of the MD simulation. LsAMP-1 is depicted as ribbons (left-side,) and in terms of its hydrophobic surface (right-side), which is colored from red to blue according to hydrophobicity, where red represents hydrophobic residues. In both cases, hydrogen bonds are marked with dashed lines. At the bottom, a root mean square deviation (RMSD) plot of the defensin mean backbone variation against heavy atoms of PIP2 across 200 ns of MD simulation (left-side); and a 2D representation of LsAMP-1/PIP2, indicating hydrogen bonds (dashed lines) and hydrophobic interactions (green lines) (left-side)

Once in hands with high-quality models of these defensins, the next move was to evaluate possible cellular ligands, which was performed by combining data from structural, molecular docking, and MD simulations. Although clear candidates could not be identified for LsAMP-2, comparative structural analysis using *Cofactor* indicates that LsAMP-1 may likely bind to phosphatidylinositol 4,5-bisphosphate (PIP2), with a BS-score of 1.10, within a surface neighboring the γ-core motif (Table 3; Fig. 3). This data was further confirmed through molecular docking, where the best binding pose shows a strong energy affinity of -7.95 Kcal/mol (Table 3; Fig. 3); and through a 200 ns MD simulation, which indicate that, after a short period (5 ns) of molecular accommodation, LsAMP-1 and PIP2 remained stably connected by two hydrogen bonds, linking Lys-39 and Asn-3 residues to PIP2 bisphosphate and by hydrophobic interactions between Phe-8 and Phe-10 residues and the fatty acid tail until the end of the run (Fig. 3).

**Table 3:** Predicted cellular ligand of LsAMP1 and NAD1

	Cofactor					DockThor	
**Model**	**Predicted ligand**	**PDB hit**	BS-score*	**Identity**	**Coverage**	**Binding energy (-Kcal/mol)**	**Hydrogen bonds**
LsAMP-1	PIP2	4cqkA	1.10	0.21	0.82	7,950	Lys39, Asn3

*BS-score is a measure provided of local similarity between template and predicted binding site in the defensin

## Discussion

CS-αβ defensins are ancient molecules, which may have evolved from bacterial cysteine-stabilized α-helical motifs [31] and emerged, according to the current knowledge, at the dawn of eukaryotic cell evolution, in a putative common ancestor of plants, fungi, and animals [32]. AMPs are effective against a wide variety of pathogens through different mechanisms of action [21] and exhibit low cytotoxic effects to human cells, are thermally and proteolytically stable, amenable to rational engineering, and rarely induce acquired-resistance in comparison to conventional antibiotics [21, 33]. Additionally, many CS-αβ defensins, such as lucifensin [34], isolated from the blowfly *Lucilia sericata*, and scedosporisin [35], isolated from the fungus *Scedosporidium apiospermum* are highly effective against methicillin-resistant *Staphylococcus aureus* and vancomycin-resistant Enterococci, respectively, highlighting these molecules have great potential to serve as alternatives to conventional antibiotics in human therapeutics. However, different methodological challenges still limit their practical pharmacological applications [36], such as low yields after purification procedures from natural hosts; and misfolding, degradation, and toxicity issues when total synthesis and/or heterologous expression approaches are applied [36].

Here, two novel CS-αβ defensins were characterized from a freshwater spirotrich ciliate, *Laurentiella* sp., representing the first report of such molecules in protists. Moreover, many ciliates, *Laurentiella* sp. included, are culturable under in vitro conditions, producing dense cultures in short time spans; and are known to synthesize a variety of antibacterial, antifungal, and antiviral compounds [37–40]; It is important to highlight the great and underexplored biotechnological potential these organisms as sources of novel bioactive compounds for human therapeutics.

LsAMP−1 and LsAMP−2 are clearly homologs of heliomicin and ARD1, two CS-αβ defensins isolated from lepidopteran insects. Besides the observed high amino acid sequence identity, both LsAMP-1 and LsAMP-2 also display the same pattern of structural cysteines (C_2_-C_6_, C_3_-C_8_, C_4_-C_9_) that is characteristic of insect defensins (SCOP:4,003,175), according to the ten cysteine reference array system proposed by Ellen Tarr [41], in which have been used to classify sequences within the CS-αβ superfamily, also known as scorpion toxin-like superfamily (SCOP:3,000,309).

Because of the great phylogenetic distances between ciliates and insects, the existence of insertion elements and/or insect genes in the vicinity of these sequences was tested. However, any sign of horizontal gene transfer events or insect DNA contamination could be noticed (data not shown), suggesting these pronounced similarities may be a consequence of strong selection pressures. Moreover, LsAMP−1 and LsAMP−2 were identified from the same host and are also greatly similar to each other (Fig. 1), raising the hypothesis that they may have evolved through duplication followed by diversification events.

Most CS-αβ defensins (including heliomicin and ARD1) characterized so far are cationic, which is often invoked to explain their selectiveness against negatively charged membranes of bacteria and fungi [42]. On the other hand, existence of many anionic CS-αβ defensins [43–46], such as LsAMP-1 and LsAMP-2 (net charge of -1 and − 5 at pH 7.0, respectively), and AfusinC isolated from *Aspergillus fumigatus*, which is active against different multi-resistant humans bacterial pathogens [47], stress that electrostatic interactions may not be enough to describe the diversity of membrane-CS-αβ defensin associations.

Structural, sequence, and phylogenetic analyses suggest that LsAMP-1 and LsAMP-2 might be true defensins with fungicide activity (Table 1; Fig. 1). This data should be confirmed through future in vitro testes, but bring new perspectives and possibilities for the control of common resistant and multiresistant humans pathogens, such as *Aspergillus fumigatus* and *Candida auris*, which figures in the 2019 US CDC’s antibiotic resistance threats report (https://www.cdc.gov/DrugResistance/Biggest-Threats.html).

CS-αβ defensins may exert their functions through many different mechanisms of actions, which include depolarization and pore formation within targeted plasma membranes, but also could attack intracellular components, such as ones involved in protein synthesis [21]. Sequence and structural analyses performed here, indicate that LsAMP−1 may interact with PIP2, which is a rare, but essential phospholipid of eukaryotic plasma membranes, found in the inner leaflet, where it is involved in actin organization, membrane trafficking and ion channel regulations [48], and in the outer leaflet, with role in cell adhesion and motility [49]. In fact, it was previously characterized that the plant defensin NAD1 can interact with PIP2, first as a dimer, then forming an oligomeric arrangement in the plasma membrane of fungi and tumor cells, in a process that culminate with membrane permeabilization, through blebs formation followed by membrane rupture, possibly involving the disruption of cytoskeleton-membrane interactions [50]. From the results obtained, one cannot rule out similarities between the mechanism of action of NAD1 and LsAMP-1, however, experimental data, such as from crystallography or Cryo-TEM would be required to evaluate this hypothesis. In any case, the best binding pose assumed by the complex, with the hydrophobic region of LsAMP-1 oriented toward PIP2 long fatty acid tail, suggests a mechanism for its anchorage into the plasma membrane of targeted cells.

## Conclusion

This study stresses the importance of public sequence databases and computational-based methods in drug discovery, offering a rapid, efficient, and low-cost framework for the identification of new antimicrobial agents. The two CS-αβ defensins (LsAMP−1 and LsAMP−2) characterized here were identified from a fast-growing freshwater ciliate, which amenable for in vitro culturing, stressing the biotechnological potential of these protists, which could become a new source of biomedical molecules.

## Methods

### Genomic screenings for CS-αβ defensins homologs

The 23 ciliate genomes analyzed in this study were directly retrieved from the NCBI nucleotide database [51]. Open reading frames (ORFs) were identified and extracted using *getorf* [52], applying proper genetic codes for each ciliate species, as suggested through analyzes using *facil* [53]. Since CS-αβ defensins are typically small proteins, only ORFs coding for proteins within 10–100 aa were considered in this analysis, which were performed using two combined approaches: homology search, using *BlastP* [54], against the SwissProt database [55] applying an e-value cutoff of 10 ^− 5^; and by pattern search, using *fuzzpro* [52] with the previously described CS-αβ defensin profile (CX2−18CX3CX2−10[GAPSIDERYW]X1CX4−17CXC, where C is for cysteine, X, stands for any amino acid residue, the subscript values are the range of occurrence and the square brackets delimit an ambiguous region in which only one of these residues can be find in that position) [27]. Next, candidates containing signal peptides - *SignalP* v5.0 [56] - (which were removed subsequently), no transmembrane domains - *Phobius* v1.01 [57] - and presenting conserved CS-αβ defensin secondary structures (αββ or βαββ) - *Psipred* [58] were selected and tested for antimicrobial activities by using 3 different AMPs predictors: *ADAM* [59], *CAMP* [60], and *iAMP−2 L* [61]. In this framework, only sequences unanimously classified as AMPs were selected for further characterizations to reduce false-positives.

### Protein 3D structure prediction

Templates for comparative three-dimensional (3D) modeling of LsAMP−1 and LsAMP−2 were selected from Protein Data Bank (PDB) (https://www.rcsb.org/), using *Hhpred* [62]. Then, 100 models for each protein were constructed using *Modeller* v9.19 [63], and the structure with the lowest DOPE (Discrete Optimized Protein Structure) score was selected as the best model for each defensin. These models were quality checked using *ProSa* II [64] and *MolProbity* [65] and visualized using *PyMOL* v2.1 (https://pymol.org/2/).

### Phylogenetic analyses

*Dali* server [66] was used to perform pairwise structural alignments of LsAMP−1 and LsAMP−2 with protein structures available in PDB; and neighbor-joining trees were subsequently inferred using these alignments with *SeaView* v4.6 [67].

### Molecular dynamics

Molecular dynamics simulations with LsAMP−1 and LsAMP−2 were performed using *Gromacs* v5 [68] applying the all-atom force field OPSL [69]. First of all, models were minimized using the steepest *descent* algorithm, centered in dodecahedral boxes with sides of 2.0 nm filled with water molecules that were modeled with TIP3P (TIP 3-point) and their geometry constrained with SETTLE algorithm [70]. Then, systems were neutralized with 0.15 mol/L of sodium chloride and after applying position constraints, equilibrated using NVT followed by NPT ensembles, considering a temperature of 300 K and pressure of 1 bar, both for 100 ps. All-atom bond lengths were linked using the *LINCS* algorithm [71]; electrostatic interactions were treated with the particle-mesh Ewald (PME) method [72], considering the electrostatic and van der Waals cutoffs of 1.0 nm, updating the list of neighbors of each atom every 10 simulation steps of 2 fs. Finally, models were released from their position constraints and production was performed for 1 µs using the *leap-frog* algorithm as the integrator. These MD simulations were analyzed by means of root-mean-square deviation (RMSD), using the backbones as units for root square calculations, rot mean square fluctuation (RMSF) and by means of Dictionary of Protein Secondary Structures (DSSP), using rms, rmf, and do_dssp available from *Gromacs* package, respectively.

### Ligand prediction

To shed some light into the mechanism of action of these defensins, comparative structural analysis were performed, using *Cofactor* [73], in which data were further confirmed through molecular docking and MD simulations. Predicted potential ligands suggested during *Cofactor* analysis were docked to the predicted binding site within the defensins, using *DockThor* web-server (https://www.dockthor.lncc.br/v2/). The 2D representations of all tested ligands were retrieved from *PubChem* (https://pubchem.ncbi.nlm.nih.gov/) and *obabel* [74] was used to convert them to 3D structures. Next, protonation states of these defensins were automatically predicted using H++ (http://biophysics.cs.vt.edu/H++), applying pH = 7.5, octahedral solvent box, TIP3P water model, and neutralization with NaCl. Finally, Charmm force field parameters of potential cellular ligands, were generated with *SwissParam* (https://www.swissparam.ch/), then, predicted defensin-ligand pairs were subjected to 200 ns MD simulations using all-atom force field Charmm27 [75] and following the procedure described in the previous section. Binding pose 2D representations were done using Pose2view (https://proteins.plus/).

## Electronic supplementary material

Below is the link to the electronic supplementary material.

Supplementary Material 1

## Data Availability

The datasets used and/or analyzed during the current study are available from the corresponding author on reasonable request.
