# Supplementary figures and images for: In silico characterization of cysteine-stabilized αβ defensins from neglected unicellular microeukaryotes
Source: BMC Microbiol. 2023 Mar 25;23:82. doi: 10.1186/s12866-023-02817-w (PMC10040121; doi:10.1186/s12866-023-02817-w)

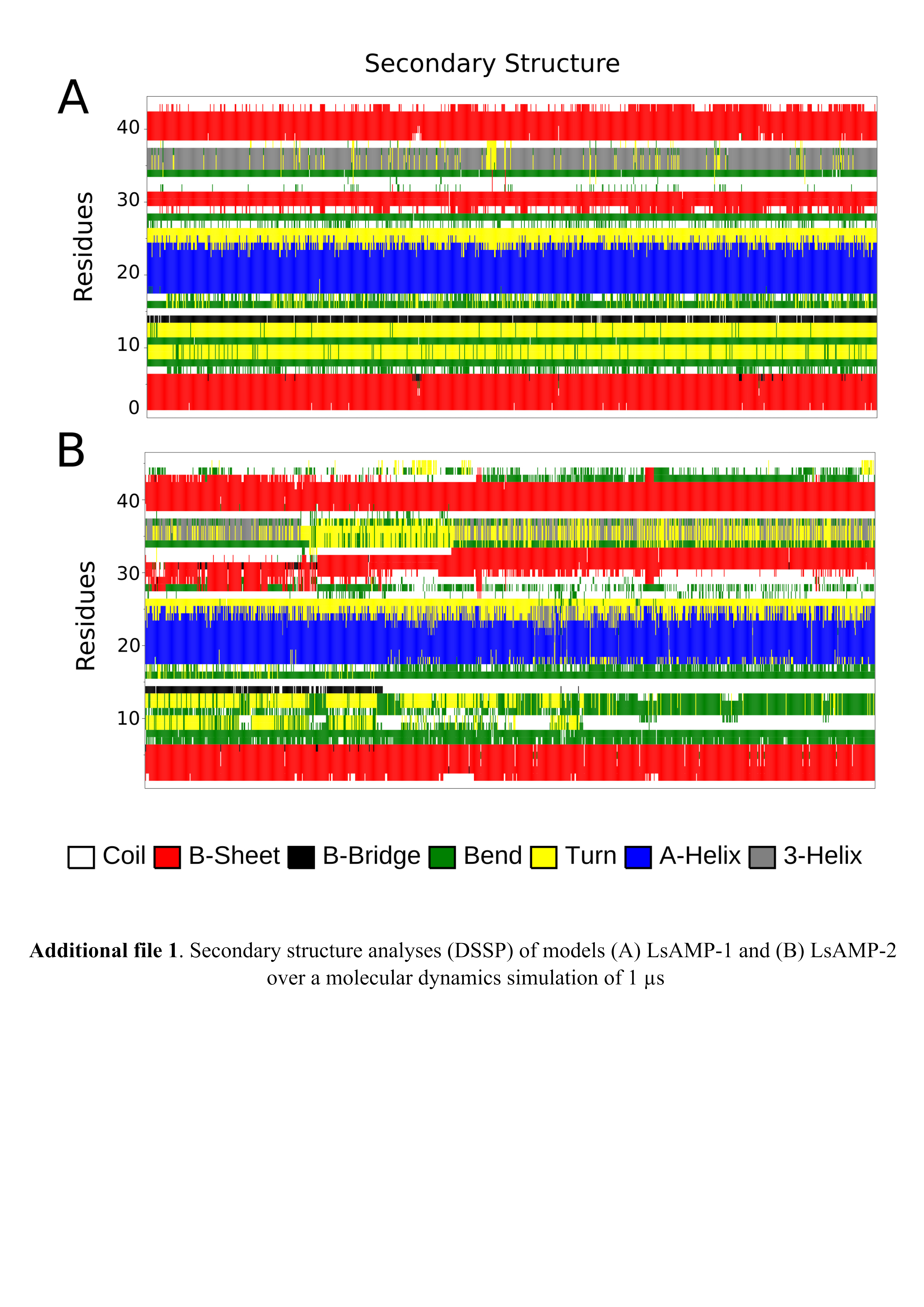

Supplement: Supplementary file 1 — Supplementary Material 1 [file 12866_2023_2817_MOESM1_ESM.jpg]
